# Research on the Corrosion Resistance and Mechanical Properties of Graphene Oxide–Modified AT13 Coatings

**DOI:** 10.3390/ma18102168

**Published:** 2025-05-08

**Authors:** Yuchen Xu, Zhenhua Chu, Jingxiang Xu, Wan Tang, Li Gao

**Affiliations:** Department of Mechanical Engineering, College of Engineering, Shanghai Ocean University, Shanghai 201306, Chinajxxu@shou.edu.cn (J.X.); wtang@shou.edu.cn (W.T.); lgao@shou.edu.cn (L.G.)

**Keywords:** graphene oxide, Al_2_O_3_-13%TiO_2_, corrosion resistance, fracture toughness, thermal shock resistance

## Abstract

The ongoing development of maritime powers has driven markedly growing requirements for novel naval and civilian vessel categories in recent years. The import temperature of gas turbines is rising, and the issue of corrosion can no longer be ignored, creating an urgent need to develop coatings with high-temperature resistance, corrosion resistance, and good toughness. This study utilized plasma spraying technology to prepare composite AT13 ceramic coatings with 0 wt.%, 5 wt.%, 10 wt.%, and 15 wt.% GO/Cu (GO:Cu = 1:10) content. It systematically investigated the effects of GO/Cu doping on the porosity, Vickers hardness, fracture toughness, thermal shock resistance, and corrosion resistance of the AT13 coatings while exploring the corrosion behavior of the composite coatings. The experimental results indicate that doping with GO/Cu can effectively fill the pores of the coatings, leading to an overall improvement in coating performance. The coating with 10 wt.% doping (G2) exhibited the best comprehensive performance, with a 72% reduction in porosity compared to the original coating, a 23.2% increase in Vickers hardness, a 31.4% enhancement in fracture toughness, and an 83% decrease in corrosion rate. It also demonstrated the best thermal shock resistance, maintaining a relatively intact surface after 31 days of immersion in artificial seawater, with only a few pitting and cracking defects observed in the areas of corrosion.

## 1. Introduction

The ongoing development of maritime powers has driven markedly growing requirements for novel naval and civilian vessel categories in recent years. With the continuous advancement of marine vessel performance requirements, gas turbines are gradually replacing traditional steam and diesel power systems due to their advantages of high thermal efficiency, smooth operation, and environmental friendliness. However, the increasing inlet temperatures of gas turbines have led to prominent issues, including increased tip clearance caused by thermal erosion, deterioration of compressor performance, and failure of turbine blades due to fracture [1,2]. Traditional coating protection systems can no longer adapt to gas turbines’ increasingly high operating temperatures [3]. Nowadays, thermal barrier coatings are often selected to withstand high-temperature conditions. Conventional thermal barrier coatings, such as yttria-stabilized zirconia (YSZ), Al_2_O_3_-TiO_2_, and rare earth zirconates, are primarily porous coatings that can operate effectively at temperatures exceeding a thousand degrees Celsius [4,5,6,7,8]. Under high-temperature marine environments, the toughness of coatings is subject to the coupled effects of chemical corrosion, mechanical loads, and wet-heat aging [9,10], which pose severe challenges to the toughness and corrosion resistance of the coatings. Existing thermal barrier coatings, which are primarily ceramic-based, exhibit poor toughness, and their porous structure is prone to accumulation of corrosion products, leading to the formation of micro-cracks and local delamination in the coatings, rendering them inadequate to meet current usage demands. Developing coatings that are high-temperature, corrosion-resistant, and tough is crucial for increasing the service life of marine engineering equipment, particularly gas turbines.

To enable existing thermal barrier coatings to adapt to the increasingly harsh operating conditions of gas turbines, it is necessary to further enhance their toughness and corrosion resistance. Doping modification is a commonly used method for modifying coatings [11,12]. Sanket Mehar et al. [13] sprayed Al_2_O_3_-3 wt.% TiO_2_ (AT) coatings with different proportions of Y_2_O_3_-stabilized zirconia (YSZ) on the 316 L stainless steel substrate. The study found that the AT coating with 30% YSZ exhibited a lower friction coefficient, significantly improving its wear resistance. Among a series of doping phases, graphene oxide has become a key additive for the next generation of high-performance composite materials due to its excellent toughening effects and corrosion resistance. Usha Pandey et al. [14] incorporated graphene oxide–modified TiO_2_ into nickel-based coatings and sprayed them onto low-carbon steel. The research demonstrated that the corrosion resistance of the composite coatings was significantly improved compared to the original coatings. A. Amudha et al. [15] added graphene oxide to alumina coating and sprayed it onto the low-carbon steel substrate IS2062 [16] grade B steel, resulting in a significant enhancement in both the fracture toughness and microhardness of the coatings compared to the original coatings. Cheng Zhang et al. [17] prepared thermoplastic polyurethane (TPU) composite materials with different compositions. The study indicated that the composite filler with an Al_2_O_3_ content of 8.9 wt.%, a GO content of 0.1 wt.%, and a Si_3_N_4_ws content of 1.0 wt.% exhibited excellent thermal stability and toughness. Current research often focuses on one or two aspects, such as high-temperature resistance, corrosion resistance, and high toughness, which do not meet the demands of marine gas turbines for high temperature resistance, high salinity resistance, and high toughness [18,19,20,21,22,23].

This study addresses the requirements of marine gas turbines for high temperature resistance, high salinity resistance, and high toughness by preparing composite AT13 coatings with different GO/Cu contents using plasma spraying technology. The effects of GO/Cu addition on the corrosion resistance, toughness, and thermal shock performance of the composite coatings were investigated through characterization, aiming to identify the optimal composition and explore the corrosion behavior in artificial seawater.

## 2. Materials and Methods

### 2.1. Experimental Raw Materials

The substrate selected is 45# steel, which was cut into dimensions of 10 mm × 12 mm × 10 mm for subsequent use. Before spraying the coating, the substrate must undergo sandblasting and dust removal treatment. The coating material AT13 powder and the bond layer spraying material NiCrAlY were both purchased from Beijing Sanspu Rui New Materials Co., Ltd. (Beijing, China); the additive material Cu powder was purchased from Shanghai National Nano Technology Co., Ltd. (Shanghai, China), and the GO was sourced from Suzhou Carbonfeng Graphene Technology Co., Ltd. (Suzhou, China). The specific parameters of the experimental materials are shown in Table 1.

### 2.2. Preparation of Coatings

**Figure 1:**
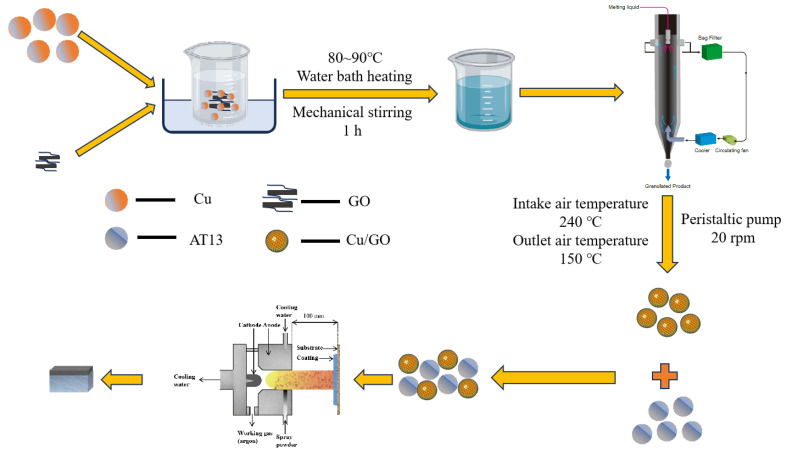
Schematic diagram of the coating preparation process.

**Figure 2:**
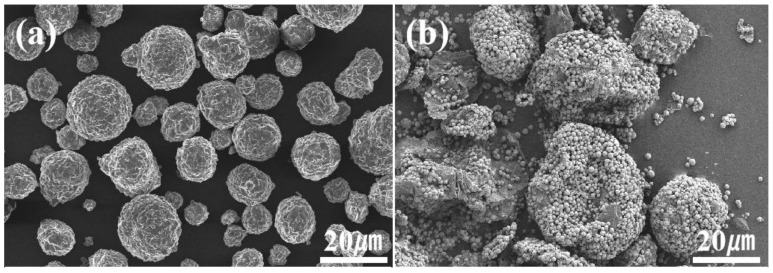
SEM morphology of AT13 and GO/Cu powders: (**a**) AT13; (**b**) GO/Cu composite powder.

This study strictly followed the procedure outlined below. The schematic diagram of the coating preparation process is shown in Figure 1. First, the GO powder has a small particle size and cannot be used directly for spraying. Therefore, GO powder was mixed with Cu in a ratio of 1:10 and agglomerated using spray granulation to prepare a GO/Cu composite powder suitable for spraying. The composite powder was then blended with AT13 powder in a mixing machine for 12 h to obtain a uniformly mixed composite AT13 powder. The microscopic morphology of the selected powder is shown in Figure 2. To find the appropriate doping ratio, composite spray coating powders with GO/Cu contents of 5 wt.%, 10 wt.%, and 15 wt.% were prepared.

Due to the significant difference in thermal expansion coefficients between the ceramic coating and the substrate, a bonding layer was added between them. Using plasma spraying technology, a bonding layer was first applied to the substrate’s surface, followed by spraying of the original powder and composite powders with three different contents onto the bonding layer. Table 2 shows the spraying parameters. The prepared coatings are classified into four types––G0, G1, G2, and G3––according to the proportion of GO/Cu composite powder, corresponding to AT13 composite coatings with GO/Cu composite powder contents of 0 wt.%, 5 wt.%, 10 wt.%, and 15 wt.%, respectively.

### 2.3. Characterization Analysis Methods

**Figure 3:**
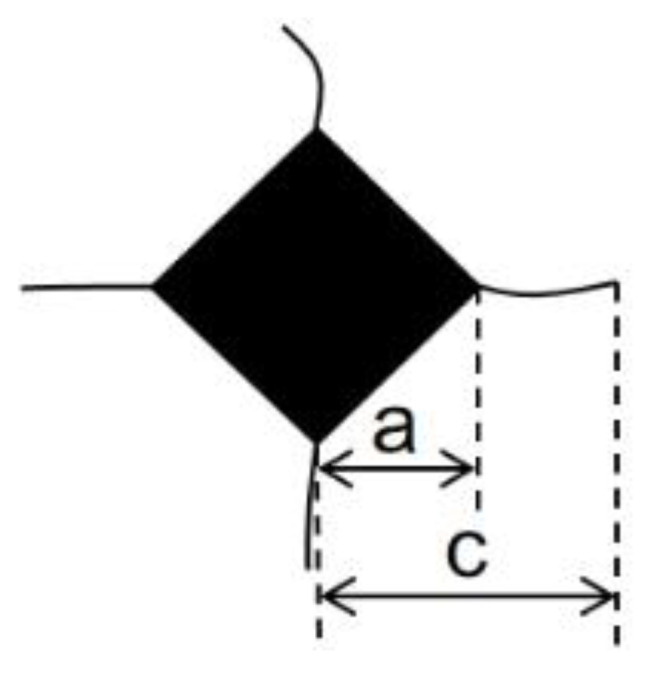
Schematic diagram of the indentation method.

The surface morphology of samples was observed using a scanning electron microscope (SEM, JSM-7500F, JEOL, Tokyo, Japan), and energy dispersive spectroscopy (EDS) analysis was performed. The porosity test was conducted by calculating the proportion of pores in surface and cross-sectional morphology images of the coating obtained via SEM using ImageJ software (https://imagej.net/ij/ (accessed on 1 September 2023)). The coating’s hardness was tested with a micro Vickers hardness tester at a loading pressure of 3 N for a holding time of 15 s. Twelve data points were measured to calculate the average value. The fracture toughness was calculated using the indentation method, and Figure 3 illustrates the schematic of the indentation method. The formula for calculating fracture toughness [24] is:

(1) $$K_{IC}=0.16HV\sqrt{a}{\left(\frac{c}{a}\right)}^{\frac{3}{2}}$$

In this context, $K_{IC}$ represents the fracture toughness (MPa·m^¹/²^), HV denotes the Vickers hardness (GPa), a is half of the diagonal length of the indentation (μm), and c is the distance from the center of the indentation to the tip of the crack (μm).

Thermal shock performance was tested in a tubular resistance furnace. During the testing, the furnace temperature was raised to 800 °C, and the sample was placed inside to heat for 5 min, followed by a 20 min hold. Afterward, the sample was quickly removed and immersed in water for cooling. When the temperature dropped to 30–50 °C, this signified completion of one thermal shock experiment. This process was repeated until failure occurred (when visible macroscopic cracks appeared on the surface or the area of local delamination exceeded 10% of the overall area) to test the thermal shock performance of the coating. The coefficient of thermal expansion (CTE) of the composite coating was estimated using the rule of mixtures:

(2) $$\alpha_{composite}=\phi_{AT13}\alpha_{AT13}+\phi_{dopant}(0.9\alpha_{Cu}+0.1\alpha_{GO})$$

In this context, *ϕ_AT_*_13_ and *ϕ_dopant_* are the volume fractions of the matrix and the dopant, respectively, *α_AT_*_13_ = 8.5 × 10^−6^/K [25], *α_Cu_* = 16.5 × 10^−6^/K [26], and *α_GO_* = 6.0 × 10^−6^/K [27].

The electrochemical tests were conducted using an electrochemical workstation (Reference 600+, Gamry, Warminster, PA, USA). In this setup, a classic three-electrode system was employed. The composite ceramic coating sample served as the working electrode, while the counter electrode and reference electrode were a platinum grid and a saturated calomel electrode (SCE), respectively. A 3.5% NaCl solution was used as the electrolyte in the electrochemical tests. Polarization scans were performed at a scan rate of 1 mV/s, ranging from −0.5 mV to 0.5 mV. Before all tests, the samples were exposed to the electrolyte solution for 1 h to achieve a stable open-circuit potential. The polarization curve parameters were analyzed using the Tafel extrapolation method. In order to further study the corrosion failure process of the composite coatings and the corrosion law, the G2 coating, which performed better in the polarization curves and has a more stable coating structure, was selected to undergo a 31-day total immersion experiment in artificial seawater (Table 3 presents the formulation of artificial seawater). The electrochemical impedance spectra of the G2 coating at 1, 4, 10, 18, 24, and 31 days were measured through an electrochemical workstation, and the samples that had been immersed for 31 days were finally tested for their corrosion mechanism.

## 3. Results and Discussion

### 3.1. Morphology and Porosity

**Figure 4:**
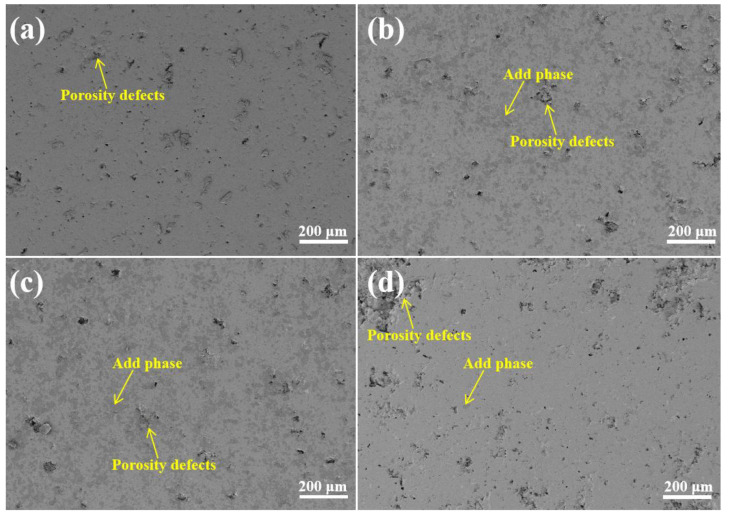
Schematic diagram of coating surface morphology: (**a**) G0; (**b**) G1; (**c**) G2; and (**d**) G3.

Figure 4 shows the surface microscopic morphology of the G0, G1, G2, and G3 composite coatings formed by plasma spraying. As can be seen from the figure, the presence of additive phases was observed on the surface of the G1, G2, and G3 coatings. The additive phases of the G1 and G2 coatings were uniformly distributed, with fewer defects; the additive phases of the G3 coating exhibited uneven phase distribution and had a greater number of porosity defects compared to the original coating.

**Figure 5:**
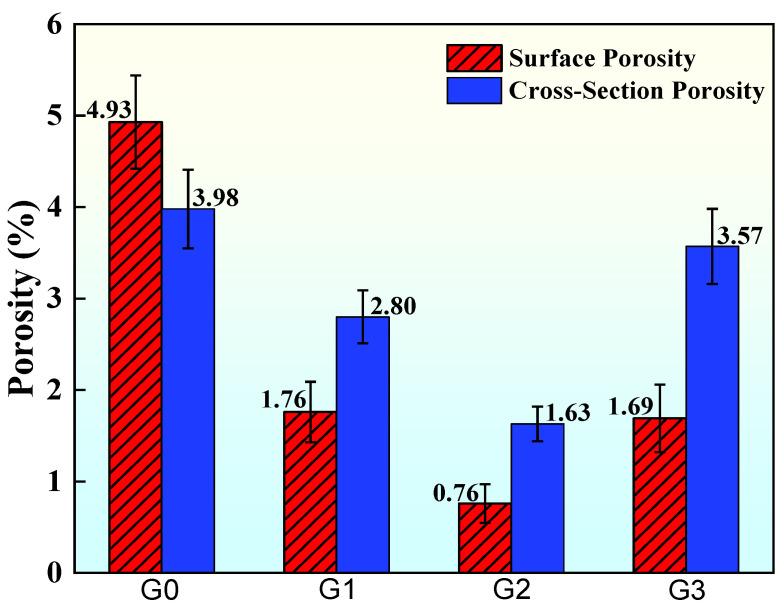
Schematic diagram of coating porosity.

The porosities of the coating surface and cross-section, as shown in Figure 5, were calculated from SEM images using ImageJ software. The figure shows that the coating porosity increased successively with the addition ratio of GO/Cu. When GO/Cu was added in smaller amounts, it melted into a flat shape at high temperatures and stayed inside the coating to fill the internal pore defects, making the coating denser. A larger amount of GO will accumulate on the surface of the substrate due to its higher melting point, further causing defects [28].

**Figure 6:**
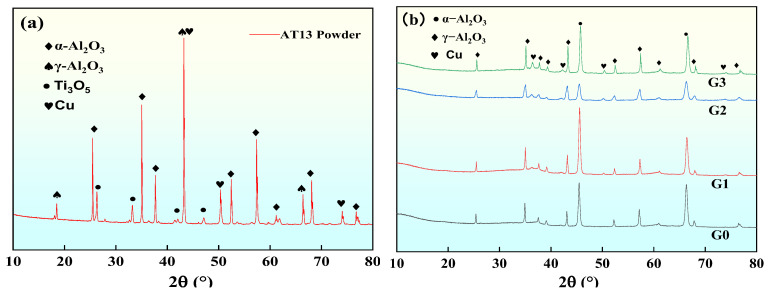
XRD spectra of the coating: (**a**) AT13 powder; (**b**) AT13 coating.

Figure 6a,b show the XRD patterns of AT13/GO/Cu powder and the four coatings, respectively. The AT13 composite powder consists of α-Al_2_O_3_, γ-Al_2_O_3_, Cu, and Ti_3_O_5_ phases, while the coating consists of α-Al_2_O_3_, γ-Al_2_O_3_, and Cu phases. The diffraction peaks in the AT13 composite coating predominantly correspond to γ-Al_2_O_3_, with only a minority attributed to α-Al_2_O_3_. This phenomenon occurs because the nucleation capability of various phases in the melt is determined by the critical nucleation free energy of the solid phase, the critical free energy of γ-Al_2_O_3_ is lower compared to that of α-Al_2_O_3_, and γ-Al_2_O_3_ is preferentially nucleated when the powder is deposited as a melt on the surface of the substrate [29].

The formation of the α-Al_2_O_3_ phase is primarily due to incomplete melting of the powder during the plasma spraying process, the presence of semi-molten particles, and the localized high temperatures caused by the spraying operation, which transformed part of γ-Al_2_O_3_ into the α-Al_2_O_3_ phase [30]. Figure 6b shows that G0, G1, and G3 have more prominent α-Al_2_O_3_ phases, while G2 has fewer α-Al_2_O_3_ phases. The appearance of the TiO_2_ phase was not found in the XRD patterns of each coating, which was attributed to the fact that TiO_2_ might partially react with Al_2_O_3_ to form a solid solution during the plasma spraying process, resulting in weakening or disappearance of the pristine TiO_2_ phase [31,32].

**Figure 7:**
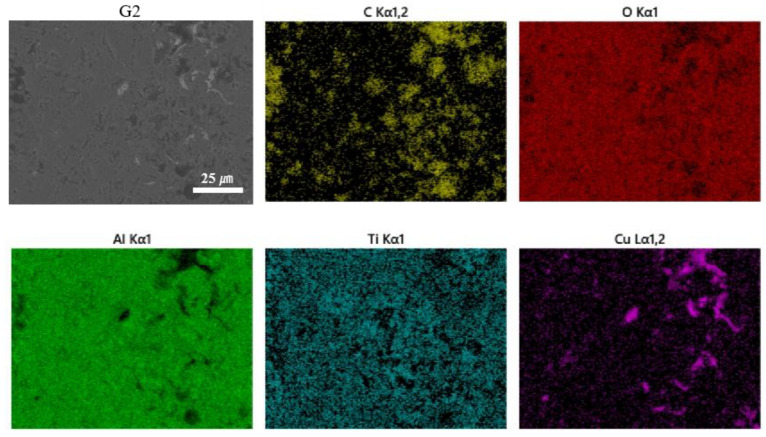
EDS spectrum of the G2 coating.

To confirm the presence of TiO_2_ in the composite coating, an EDS spectrum analysis was conducted on the G2 coating. Figure 7 shows the spectrum of the G2 coating, which clearly indicates the presence of titanium (Ti) with a uniform distribution, thereby confirming the existence of TiO_2_.

### 3.2. Vickers Hardness and Fracture Toughness

**Figure 8:**
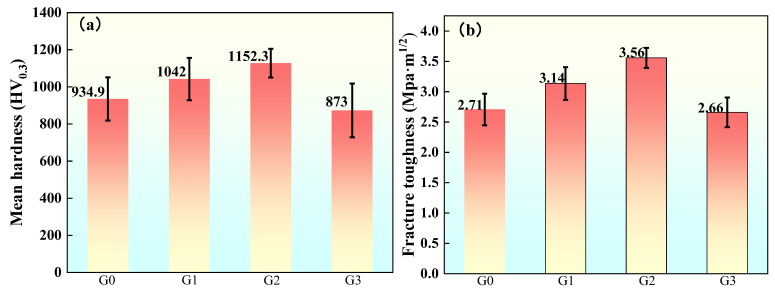
Mechanical properties of the coatings: (**a**) Vickers hardness; (**b**) fracture toughness.

Figure 8a presents the micro Vickers hardness profile of the coatings. As evident from the data, the Vickers hardness initially increased then decreased with GO/Cu addition, with sample G2 exhibiting a peak hardness of 1152.3 HV_0.3_, representing a 23.2% enhancement compared to the baseline coating G0. Excessive addition of GO/Cu significantly reduced the Vickers hardness of the coating. The G3 coating, which contains 15% GO/Cu, exhibited a 6.7% decrease in Vickers hardness compared to the original coating.

Figure 8b shows the fracture toughness of the coatings calculated by the indentation method. The addition of GO/Cu significantly improved the fracture toughness of the coatings, and the trend was the same as that of the Vickers hardness. Doping with GO/Cu comprehensively improved the mechanical properties of the coatings.

When a small amount of GO/Cu is added to the AT13 coating, because the specific surface area of GO is large enough to be sufficiently coated with the AT13 coating matrix, there is enough surface transfer stress between GO and the coating matrix to transfer the loads on the coating to GO. Because GO has a high Young’s modulus (Young’s modulus of monolayer graphene can reach up to 231 GPa [33]), the strength of the composite material was significantly improved during the load transfer. Cu, as the carrier of GO, has a low melting point. It can fully melt and further fill the pores and surface defects during the spraying process to enhance the coating’s performance. However, when the amount of GO is excessive, this can lead to aggregation and accumulation. During the spraying process, this causes the coating to exhibit a higher number of casting defects (such as porosity and impurities), thereby reducing the strength of the coating [34,35,36]. In this study, the G2 coating with a 1% GO content had mechanical properties that increased its Vickers hardness by 23.2% and fracture toughness by 31.4% compared to the original coating.

### 3.3. Thermal Shock Performance

**Figure 9:**
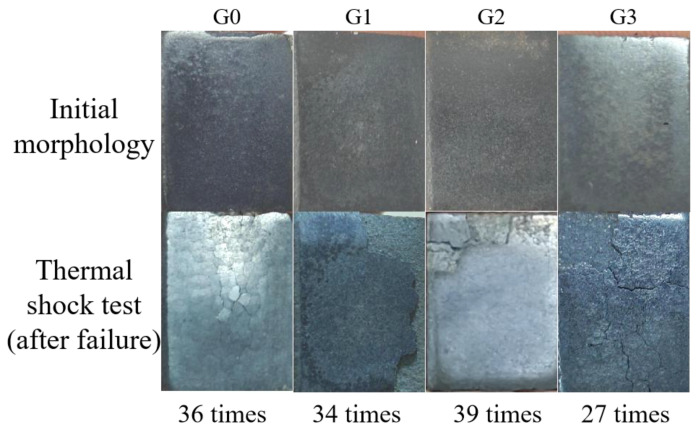
Macro morphology of the coatings before and after thermal shock.

As shown in Figure 9, the AT13 composite coatings exhibited distinct macroscopic morphological evolution during thermal shock testing. The undoped reference coating (G0) developed visible surface cracks detectable by eye after 36 thermal cycles, marking its failure. In contrast, the GO/Cu-doped coatings G1 and G2 showed no macroscopic cracking after 34 and 39 cycles, respectively. However, edge delamination exceeding 10% of the total coating area was observed at these stages, defining their failure criteria. Notably, coating G3 failed prematurely at 27 cycles due to extensive surface cracking concurrent with large-area spallation.

Analysis of failure modes revealed that moderate GO/Cu doping (G1, G2) enhanced thermal shock resistance, while excessive doping (G3) introduced detrimental defects. The thermal shock resistance ranking was as follows: G2 > G1 > G0 > G3, with G2 demonstrating optimal performance.

**Figure 10:**
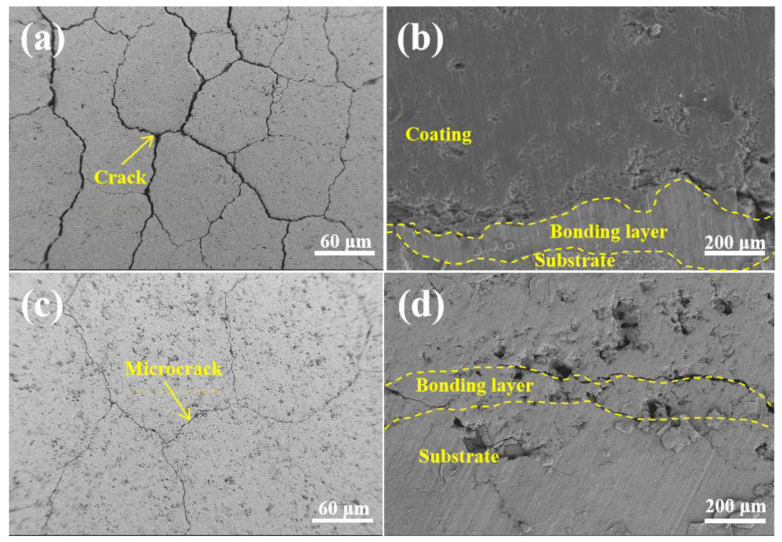
Comparison of coating surface and cross-sectional morphology after thermal shock test: (**a**) G0 surface morphology; (**b**) G0 cross-sectional morphology; (**c**) G2 surface morphology; (**d**) G2 cross-sectional morphology.

Figure 10 compares the surface and cross-section morphology of the G0 and G2 coatings after thermal shock experiments. Figure 10a,c show that more cracks were distributed on the surfaces of G0 and G2, and the crack width for G0 was much wider compared with that for G2. Only a few small cracks appeared on the surface of G2, and the incorporation of GO/Cu obviously inhibited the cracks’ emergence and prolongation. Figure 10b,d show that the G0 coating had only slight cracks between the bonding layer and the ceramic layer, while the G2 coating had obvious cracks. This is because the difference in thermal coefficients between the ceramic layer and the bonding layer was further enlarged by the addition of GO/Cu (the thermal expansion coefficients (CTEs) of G0, G1, G2, and G3 are 8.5 × 10^−6^/K, 8.85 × 10^−6^/K, 9.2 × 10^−6^/K, and 9.55 × 10^−6^/K, respectively).

Coating G0 surface failure manifested as cracks visible to the naked eye. Cracks appear and proliferate primarily because hot and cold cycles constantly generate thermal stress in the thermal shock experiment, resulting in ceramic coating deformation, which sprouts cracks and continues to expand. Failure of the GO/Cu-doped coatings G1, G2, and G3 manifested as spalling of most of the surface of the edge of the coating. The coating spalling phenomenon is affected by a variety of factors. The first is the difference in thermal coefficients between the ceramic coating and the bonding layer; in general, as the difference in thermal coefficients between the two increases, the stress generated during thermal cycling increases significantly, making the spall phenomenon more likely [37]. During the heating stage, the coefficient of thermal expansion mismatch between the ceramic top coat and bond coat induces differential expansion, with the bond coat exhibiting greater dimensional change than the top coat. The shear stress will lead to cracking and peeling at the interface, and normal stress will lead to peeling of the coating.

In the course of this experiment, no delamination was observed in the G0 coating, while the coatings doped with GO/Cu all failed in the form of coating flaking. This phenomenon may be attributed to the increased coefficient of thermal expansion mismatch between the ceramic coating and bond coat caused by GO/Cu incorporation. During the heating process, because of the poor thermal conductivity of the ceramic coating, a temperature gradient is formed inside the coating, leading to non-uniformity of the coating, and the thermal stresses generated inside can lead to cracking of the coating. On the other hand, the metal elements of the bonding layer will be oxidized at high temperatures in the middle of the bonding layer. At the beginning, the growth of TGO is very rapid, during which Al elements preferentially oxidize to form a dense Al_2_O_3_ layer. The Al_2_O_3_ layer exhibits good anti-oxidation properties, and its presence can slow down further oxidation, inhibiting the growth rate of TGO. As the experiment continues, the TGO layer grows further, and some Y elements oxidize to form Y_2_O_3_. When the content of Al elements is further depleted to a low level, Cr and Ni elements will undergo oxidation reactions to produce Cr_2_O_3_ and NiO, among others. Pore defects in the coating affect the TGO growth rate. If the surface ceramic layer is spalled during the production of TGO, the bond coat becomes exposed to the atmospheric environment, and growth of TGO will be further promoted. During the growth of TGO, interfacial stresses are constantly generated, causing cracks to appear and spread between the ceramic layer and the TGO layer. Overall, spalling and cracking of the ceramic coating are mainly due to an excessive difference in thermal coefficients between the ceramic layer and the bonding layer, the thermal stresses generated during the ceramic layer’s warming process, and the coupling of interfacial stresses continuously generated during growth of the TGO layer.

### 3.4. Corrosion Resistance

**Figure 11:**
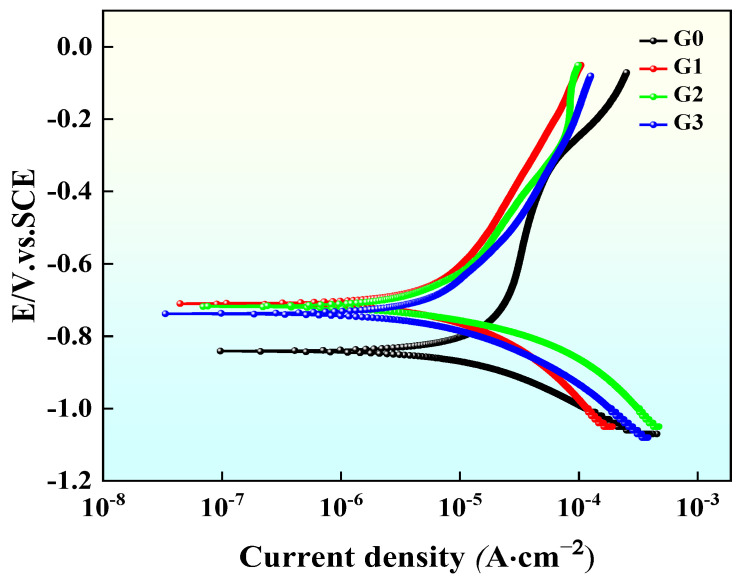
Potentiodynamic polarization curves of the G2 coating in a 3.5 wt.% NaCl solution.

The polarization curves of AT13 coatings with different GO/Cu contents in 3.5 wt.% NaCl solution were measured using an electrochemical workstation and are shown in Figure 11 after Tafel curve fitting. The self-corrosion potential *E*_corr_ reacts to the ease with which a material can be corroded, and the self-corrosion current density *I*_corr_ reacts to the actual corrosion rate of the material. The larger the *E*_corr_, the more excellent the material’s resistance to corrosion, and the smaller the *I*_corr_, the better the material’s corrosion resistance [38,39,40]. As seen from the Tafel curve in Figure 11, incorporation of GO/Cu induced a significant positive shift in the corrosion potential, demonstrating markedly enhanced corrosion resistance.

Combining the polarization curve–fitted parameters is necessary to further evaluate each coating’s corrosion resistance. As shown in Table 4, which shows the data fitted from the polarization curves, the *E*_corr_ values of the GO/Cu-modified coatings were all positively shifted by more than 100 mV compared with the original coatings, and the *I*_corr_ decreased by one order of magnitude. It is worth noting that the G1 and G2 coatings had similar self-corrosion potentials, but the G2 coating exhibited a 37.3% reduction in corrosion current density compared to G1. This difference resulted in the G2 coating showing better corrosion resistance, with the corrosion rate decreasing from 0.331 mm/a to 0.057 mm/a for the original coating.

### 3.5. Corrosion Pattern of an AT13/Cu/GO Ceramic Coating in Artificial Seawater

**Figure 12:**
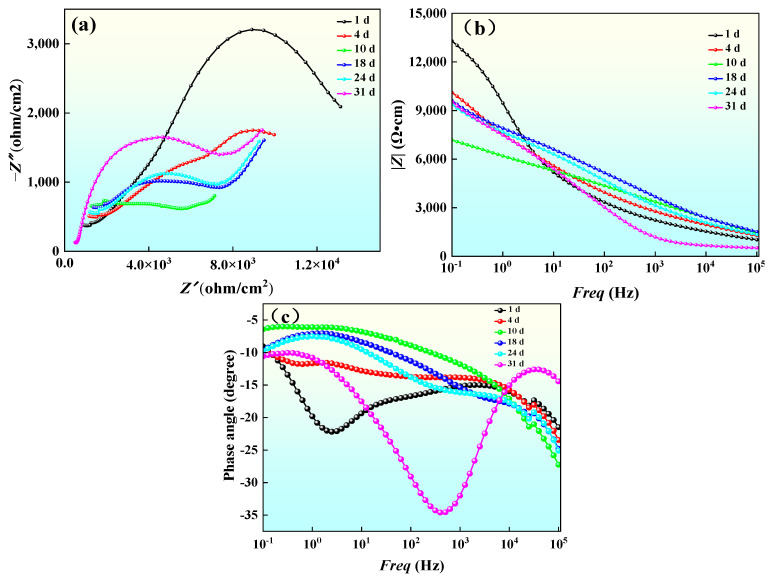
Impedance diagrams of the G2 coating immersed in artificial seawater: (**a**) Nyquist plot; (**b**) Bode phase plot; and (**c**) Bode magnitude plot.

The best-performing coating, G2, was immersed in seawater for 31 d to investigate its corrosion behavior. Figure 12 shows the impedance map of coating G2 over 31 d of immersion time. The Nyquist curve of the tolerant arc length radius size is related to corrosion resistance; the larger the radius of the capacitive arc, the more stable the passive film formed on the material surface and the better the corrosion resistance of the material [41,42,43]. The corrosion process can be categorized into two stages, pre-corrosion (1–10 days) and post-corrosion (18–31 days), based on changes in the impedance spectra of the coatings, as shown in Figure 12a. The composite AT13 coating has only a capacitive resistance arc in the pre-corrosion phase, which is mainly controlled by charge transfer. In the late stage of corrosion, the coating impedance spectrum shows a combination of capacitive arcs and Warburg diffusion curves, and this stage is mainly for the diffusion process of the coating corrosion products.

As can be seen from the Bode angular diagram, only one time constant exists in the pre-corrosion stage. In the late corrosion stage, two peaks appear in the Bode angular diagram, indicating two time constants: the first time constant corresponds to the capacitive response of the coating solution, and the second corresponds to the interfacial response of the coating substrate. The appearance of a double time constant indicates that the corrosion medium has reached the coating–substrate interface through the pores, forming a localized corrosion microcell. The Bode phase diagram, to a certain degree, reflects changes in the corrosion resistance of the coating, as can be seen from the figure; the trend of its impedance value and the Nyquist diagram has a consistent tendency to decline and then rise and then decline and then rise. In the pre-corrosion period, with the erosion of corrosive media, the coating corrosion resistance significantly decreased; from 10 d to 18 d, the coating corrosion resistance with the accumulation of corrosion products appeared to rebound; from 18 d to 24 d, during this stage of coating corrosion, corrosive media entered through the coating pore defects to reach the coating–substrate interface, and the formation of localized corrosion resulted in a decrease in corrosion resistance; after 31 d of immersion, the dissolution and generation of corrosion products reached an equilibrium state, having filled the pore defects, which blocked diffusion of corrosive media, and the coating corrosion resistance once again rebounded.

**Figure 13:**
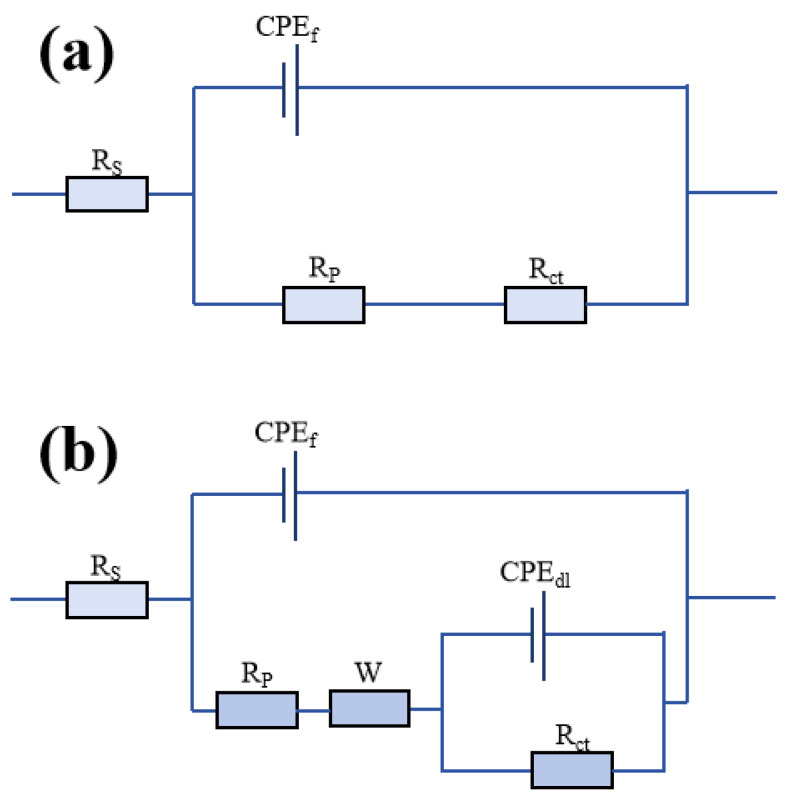
Equivalent circuit diagrams of G2 immersed in artificial seawater: (**a**) initial corrosion stage; (**b**) advanced corrosion stage.

The equivalent circuits were fitted using the software program Zview 3.1, and the pre-corrosion circuit and post-corrosion circuit diagrams are shown in Figure 13a and Figure 13b, respectively. *R*_s_ represents the solution resistance; *R*_p_ represents the pore resistance, which represents the ability of the coating to resist the intrusion of corrosive media; *R*_ct_ represents the charge transfer resistance, which can be used to express the ease of charge transfer across the interface of the two phases in the electrode process; *CPE*_f_ represents the capacitance of the coating; *CPE*_dl_ stands for the capacitance of the double electric layer, which reflects the condition of the metal surface and corrosive media; and W stands for the Warburg impedance. The statistical significance of the fitted parameters was evaluated using a threshold of α = 0.05 (95% confidence level).

The variation of the fitted parameters of each component with time is shown in Table 5. Coating capacitance *CPE*_f_ in the pre-corrosion period with the erosion of corrosive media rapidly decreased and then stabilized; during the 18–24 d period, the corrosive media underwent pore defect diffusion to reach the coating–substrate interface; and the coating capacitance further declined during the 24–31 d period due to the generation of corrosion products and dissolution of the equilibrium to rebound. The *CPE*_dl_ emerges when the corrosive medium penetrates to the coating–substrate interface, followed by a decrease due to corrosion product accumulation. Pore resistance *R*_p_ in the early stage of corrosion declined by an order of magnitude. At this corrosion stage, the medium erodes the coating through pore defects on the surface. Some tiny cracks appear on the coating. Pore defects cause severe damage to the coating by 10 d. From 10 to 18 d, *R*_p_ continuously changes with the generation and dissolution of corrosion products. After 31 d of immersion, the generation and dissolution of corrosion products reaches equilibrium. At this point, corrosion products significantly fill the coating pores. This leads to a renewed rise in *R*_p_. The charge transfer resistance (*R*_ct_) shows an overall decreasing trend. Charge transfer becomes easier. *R*_ct_ drops by an order of magnitude when the corrosive medium contacts the coating–substrate interface. Pore blockage in the later corrosion stages causes corrosion resistance to rebound. *R*_ct_ also rebounds accordingly.

**Figure 14:**
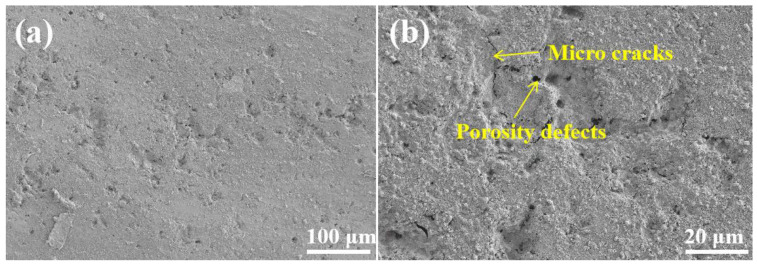
SEM images of the corrosion morphology of the G2 coating after 31 days of immersion in artificial seawater: (**a**) low magnification; (**b**) high magnification.

As seen in Figure 14a, the coating surface was relatively intact after 31 d of artificial seawater immersion, and there were no large pits or cracks. Moreover, electrochemical corrosion of the coating first appeared in the initial defective part and gradually expanded, and only a tiny number of pits and small cracks were observed after enlargement of the defective part.

### 3.6. Corrosion Failure Mechanism

**Figure 15:**
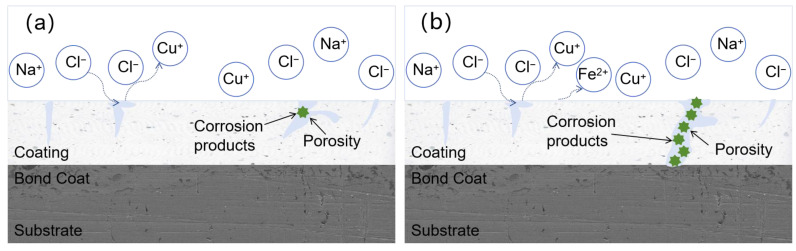
Long-term corrosion mechanism of the G2 coating: (**a**) pre-corrosion stage; (**b**) post-corrosion stage.

Figure 15 shows the corrosion mechanism of the G2 coating after soaking in artificial seawater for 31 days. After doping with GO/Cu, the coating’s porosity improved, and the surface became relatively dense without the formation of through holes. This prevented the corrosion products from directly contacting the substrate in the early stages of corrosion, allowing them to remain within the coating, thereby maintaining good corrosion resistance. As the soaking time increased, the corrosive medium diffused through the pores to the coating–substrate interface, leading to localized corrosion and a further decline in corrosion resistance. In the later stages of corrosion, a balance was reached between generation and dissolution of the corrosive medium and products, which filled the pores of the coating. The resulting blockage effect enhanced the coating’s corrosion resistance.

The addition of GO/Cu not only fills the pores of the coating, making it denser, but the flaky structure of GO, with its large specific surface area and high Young’s modulus, allows it to be dispersed throughout the coating [44,45], providing structural support. This makes the propagation of cracks within the coating more difficult, reducing the formation of corrosion pathways and significantly decreasing the corrosion rate. Additionally, its unique network structure complicates the passage of corrosive media, and graphene oxide exhibits good chemical inertia, preventing it from reacting with corrosive substances. These factors result in a convoluted path for the corrosive media within the coating, significantly extending the coating’s service life.

## 4. Conclusions

This study prepared composite AT13 coatings with different GO/Cu contents using plasma spraying technology. By characterizing the corrosion resistance, toughness, and thermal shock performance of the composite coatings, the effects of the addition of GO/Cu on the coating properties were investigated. The corrosion behavior of the composite AT13 coatings was studied through artificial seawater immersion experiments, leading to the following conclusions.

(1) Co-doping with GO/Cu promotes densification of the AT13 ceramic coating by filling the pore structure, resulting in a significant increase in its hardness. The coating G2, which was doped with 10 wt.% GO/Cu, had the lowest porosity (0.76% on the front side and 1.63% on the cross-section) and the highest Vickers hardness of 1152.28 HV_0.3_.
(2) The incorporation of GO effectively increased the coating’s toughness and inhibited crack initiation and extension under sudden temperature changes. Coating G2 had the best fracture toughness of 3.56 MPa⋅m^1/2^ and the best thermal shock performance, failing after 39 thermal shock cycles.
(3) As the GO/Cu doping ratio increases, the corrosion resistance of the coatings first rises and then decreases, and the G2 coating with a doping ratio of 10 wt.% had the best effect, with its self-corrosion current density decreasing by one order of magnitude compared to the original coating. At the same time, it had the lowest self-corrosion rate of 0.057 mm⋅a^−1^.
(4) In the 31 d artificial seawater immersion experiment, the corrosion resistance of coating G2 decreased by an order of magnitude in 0–10 d due to erosion by corrosive media; during 10–18 d, the corrosion resistance recovered due to the accumulation of corrosion products; by 18 d, the corrosive media had diffused to the coating–substrate interface through pore defects, and the corrosion resistance further decreased; during 24–31 d, the generation and dissolution of corrosion products reached an equilibrium, and the corrosion resistance recovered; after 31 d, only a small number of pits and cracks were observed on the coating’s surface.

## Figures and Tables

**Table 1:** Specific parameters of experimental materials.

Powder	Particle Size (µm)	Purity	Morphology
AT13	15~45	99.9%	Near-spherical
NiCrAlY	15~53	99.9%	Near-spherical
Cu	1~5	99.9%	Spherical
GO	0.2~10	98%	Flaky

**Table 2:** Coating spraying parameters.

Coating	NiCrAlY Bonding Layer	Composite AT13 Coating
Spraying Voltage	70 v	70 v
Spraying Current	500 A	600 A
Spraying Distance	100 mm	100 mm
Argon Gas Flow Rate	40 L/min	40 L/min
Gun Movement Speed	800 mm/s	600 mm/s
Powder Feeding Rate	40 g/min	40 g/min
Deposition Thickness	100 μm	250~300 μm

**Table 3:** Artificial seawater composition.

Reagents	NaCl	MgCl_2_	Na_2_SO_4_	CaCl_2_	KCl	SrCl_2_	NaHCO_3_	KBr	H_3_BO_3_	NaF
Dose (g/L)	24.53	5.20	4.09	1.160	0.695	0.025	0.201	0.101	0.027	0.003

**Table 4:** Fitting parameters of the polarization curve for the coatings.

	*E*_corr_ (mv)	*I*_corr_ (μA·cm^−2^)	corr Rate (mm/a)
G0	−841.2	28.47	0.331
G1	−710.3	7.905	0.091
G2	−717.9	4.958	0.057
G3	−738	6.97	0.081

**Table 5:** Equivalent circuit fitting parameters.

Time	*CPE*_f_ (F)	*R*_p_ (Ω·cm^2^)	*R*_ct_ (Ω·cm^2^)	*CPE*_dl_ (F)	Goodness of Fit
1 d	1.64 × 10^−5^	3.74 × 10^3^	1.60 × 10^4^	-	1.47 × 10^−5^
4 d	2.41 × 10^−6^	7.47 × 10^2^	7.23 × 10^3^	-	1.57 × 10^−5^
10 d	1.79 × 10^−6^	4.53 × 10^2^	3.67 × 10^3^	-	1.13 × 10^−5^
18 d	1.85 × 10^−6^	6.58 × 10^2^	3.31 × 10^3^	1.44 × 10^−5^	4.71 × 10^−4^
24 d	5.81 × 10^−7^	4.79 × 10^2^	7.5 × 10^2^	1.57 × 10^−5^	2.96 × 10^−4^
31 d	9.82 × 10^−6^	8.56 × 10^2^	9.56 × 10^3^	5.17 × 10^−6^	3.71 × 10^−4^

## Data Availability

The original contributions presented in this study are included in the article. Further inquiries can be directed to the corresponding author.
